# A domestic cat whole exome sequencing resource for trait discovery

**DOI:** 10.1038/s41598-021-86200-7

**Published:** 2021-03-30

**Authors:** Alana R. Rodney, Reuben M. Buckley, Robert S. Fulton, Catrina Fronick, Todd Richmond, Christopher R. Helps, Peter Pantke, Dianne J. Trent, Karen M. Vernau, John S. Munday, Andrew C. Lewin, Rondo Middleton, Leslie A. Lyons, Wesley C. Warren

**Affiliations:** 1 Department of Animal Sciences, College of Agriculture, Department of Surgery, School of Medicine, Institute for Data Science and Informatics, University of Missouri, Columbia, MO 65211 USA; 2 Department of Veterinary Medicine and Surgery, College of Veterinary Medicine, University of Missouri, Columbia, MO 65211 USA; 3 McDonnell Genome Institute, Washington University, School of Medicine, St Louis, MO 63108 USA; 4 Roche Sequencing Solutions, Pleasanton, CA 94588 USA; 5 Langford Vets, University of Bristol, Langford, Bristol, BS40 5DU UK; 6 AniCura Bielefeld GmbH, Tierärztliche Klinik für Kleintiere, 33719 Bielefeld, Germany; 7 Department of Biomedical and Diagnostic Sciences, College of Veterinary Medicine, University of Tennessee, Knoxville, TN 37996 USA; 8 School of Veterinary Medicine, University of California Davis, Davis, CA 95616 USA; 9 School of Veterinary Science, Massey University, Palmerston North, New Zealand; 10 Department of Veterinary Clinical Sciences, Louisiana State University, Baton Rouge, LA 70803 USA; 11 Nestlé Purina Research US, Saint Louis, MO 63164 USA

**Keywords:** Bioinformatics, Sequence annotation

## Abstract

Over 94 million domestic cats are susceptible to cancers and other common and rare diseases. Whole exome sequencing (WES) is a proven strategy to study these disease-causing variants. Presented is a 35.7 Mb exome capture design based on the annotated Felis_catus_9.0 genome assembly, covering 201,683 regions of the cat genome. Whole exome sequencing was conducted on 41 cats with known and unknown genetic diseases and traits, of which ten cats had matching whole genome sequence (WGS) data available, used to validate WES performance. At 80 × mean exome depth of coverage, 96.4% of on-target base coverage had a sequencing depth > 20-fold, while over 98% of single nucleotide variants (SNVs) identified by WGS were also identified by WES. Platform-specific SNVs were restricted to sex chromosomes and a small number of olfactory receptor genes. Within the 41 cats, we identified 31 previously known causal variants and discovered new gene candidate variants, including novel missense variance for polycystic kidney disease and atrichia in the Peterbald cat. These results show the utility of WES to identify novel gene candidate alleles for diseases and traits for the first time in a feline model.

## Introduction

Genomic medicine promises new avenues of disease treatment in veterinary medicine^1^. However, the appropriate resources are not yet readily available for robust implementation in clinical practice^2^. One resource which has been successfully applied to the diagnosis of rare diseases in humans is whole exome sequencing (WES) analysis, a cost-effective method for identifying potentially impactful DNA variants in the coding regions of genes^3^. DNA base changes in the exome can alter amino acids in proteins or disrupt their overall structure, so focusing on these regions offers a more direct and biologically interpretable approach to searching for putative disease variants. In comparison, whole genome sequencing (WGS) captures DNA variants spanning the entire genome. However, as the vast majority of the identified variants are within non-coding regions, much of the variation is difficult to interpret. The present study seeks to develop and validate the use of WES as a viable approach for determining novel disease variants in cats.

Over the last decade, a surge of studies using next generation sequencing (NGS), in particular WES, has led to many novel discoveries of candidate disease-causing variants across species. WES is recognized as an efficient means for genome resequencing and is the primary NGS approach used to help diagnose human patients with rare genetic diseases^4,5^. By selectively sequencing all protein-coding regions to a deeper depth than WGS, WES is a dependable method for finding biallelic exonic variants causative of Mendelian inherited diseases that rarely appear in healthy populations^4,5^. In humans, WES is commonly used to find genetic causes in a wide range of diseases, even complex neurological conditions such as autism spectrum disorder^6^. Its widespread use has led to the discovery of therapeutic targets for drug development and genetic markers for innovative clinical applications^7^. Tumor WES has been especially successful by cost-effectively providing somatic variant information about a patient’s normal and tumor exomes, supporting the identification of recurrent somatic mutations among known oncogenes that may suggest a mechanism of action and targets for potential drug therapies^8^. The significant depth of exome coverage is integral to overcoming diluted somatic variant allele frequencies (VAF) due to tumor clonality and purity issues.

Exome sequencing has also proven successful in non-human species. Mouse WES studies have found strong candidate alleles for models of orofacial clefting, urogenital dysmorphology, and autoimmune hepatitis^9^. In companion animals, the development of dog WES has demonstrated that causative allele discovery for common diseases has great potential^10^. Some examples in dogs include the discovery of a two-base pair deletion in *SGCD* causing muscular dystrophy, and a splice site variant in *INPP5E* which is associated with cystic renal dysplasia^11^. As there are many isolated breeds of domestic dogs, this species is an important genetic resource for cancer studies, for which WES demonstrated dogs have similar oncogene variant patterns to humans^12^. However, many oncogene variants are not equivalent to a WES analysis of human, and canine bladder cancers identified novel mutations in *FAM133*B, *RAB3GAP2*, and *ANKRD52* that are unique to canine bladder cancer, emphasizing the need to understand the biological differences in origin^13^.

Similar to canines, domestic cats have long been recognized for their potential in modeling human diseases, such as retinal blindness^14,15^. Approximately 150 variants in domestic cats are associated with over 100 genetic traits or diseases, many mimicking human disease phenotypes^16^. As feline genomic resources continue to advance, more diseases caused by single base variants are being discovered, such as two novel forms of blindness in Persian and Bengal cats^17,18^. However, a feline WES resource has not been described to date for the discovery of novel disease gene candidates. Here we describe the first feline exome resource, a WES analysis of 41 cats, and its use in the discovery of known and novel variants associated with feline phenotypes, healthy and diseased. A comparison of WES and WGS methods was also completed to understand the efficiency, depth of coverage, and sequence specificity, for variant calling from each approach.

## Results

### Phenotype cohort

WES was performed on 41 individual cats, representing a variety of different diseases and traits, some with known disease alleles (Table 1). The 41 cats can be further divided into two separate cohorts: the first is the initial ten cats that had nine known variants for various diseases and aesthetic traits, e.g., coat colors and fur types. These 10 cats also had matched WGS data, which was used to assess the efficacy of WES. The second cohort of 31 represents genetically uncharacterized cats. These cats represented 11 different breeds and include 14 random-bred cats. Groups of cats with similar genetic backgrounds were used to evaluate causes for mediastinal lymphoma, a seizure disorder, eyelid colobomas, hypothyroidism, hypovitaminosis D, blue eyes of Ojos Azules breed, and curly hair coat of the Tennessee Rex. Five cats were reported with cardiac diseases, including hypertrophic cardiomyopathy (HCM). At least seven neurological disorders are represented in the study population, generally representing novel presentations in random-bred cats. Overall, the 41 cats had approximately 31 different unknown disease presentations.

**Table 1:** Description and diseases of 41 cats for WES evaluation.

No	Id	Breed	Sex	Disease/Trait	Gene(s)
1	19725	Lykoi	F	Lykoi	*HR*
2	13230	Mixed Breed	F	Bengal PRA/Bobbed tail	*KIF3B/HES7*
3	14056	Mixed Breed	M	Persian PRA/*Long*	*AIPL1/FGF5*
4	17994	Mixed Breed	F	Hydrocephalus	*GDF7*
5	19067	Munchkin	F	Dwarfism/Dominant White	*UGDH/KIT*
6	5012	Oriental	M	Lymphoma	*Unknown*
7	20382	Peterbald	M	*Hairless*	*LPAR6*^a^
8	11615	Random Bred	M	*Dominant White*	*KIT*
9	18528	Random Bred	M	*Spotting*	*KIT*
10	20424	Siberian	F	*Long*/Cardiac disease	*FGF5/Candidate*
11	22550	Bengal	F	Polyneuropathy	*Unknown*
12	20957	Devon Rex	U	Papilloma virus	*Unknown*
13	22752	Devon Rex	M	Neurological disorder	*Unknown*
14–15	21983/21464	Ojos Azules	1M:1F	Ojos Azules	*Unknown*
16	20964	Oriental	F	Cardiac disease	*Unknown*
17	22728	Random bred	F	Cystinuria	*SLC3A1*^a^
18	20617	Random Bred	M	Neuronal ceroid lipofuscinosis	*CLN6*^a^
19	20948	Random Bred	M	Cinnamic acid urea	*Unknown*
20	21153	Random Bred	M	Ambulatory paraparesis	*Unknown*
21	22287	Random Bred	F	Myotonia congenita	*Unknown*
22	22397	Random Bred	M	Neurological disorder	*Unknown*
23	22505	Random Bred	M	Cardiac disease	*Unknown*
24	22623	Random Bred	U	Pycnodysostosis	*Candidate*
25	22740	Random Bred	F	Epidemolysis bullosa	*Unknown*
26–27	22741/22742	Random Bred	1F:1M	Eyelid coloboma	*Unknown*
28	22751	Random Bred	M	Ehlers-Danlos	*Unknown*
29–30	22763/22764	Random Bred	2F	Hypothyroidism	*Candidate*
31–32	22761/22762	Savannah	2M	Hypovitaminosis D	*Unknown*
33	21984	Scottish Fold	F	Cardiac disease	*Candidate*
34–35	20384/20385	Selkirk Rex	1F:1U	Seizures	*Unknown*
36	20953	Siamese	F	Cardiac disease	*Candidate*
37	22622	Siberian	U	PKD	*PKD2*^a^
38	22711	Singapura	F	Hypovitaminosis D	*Candidate*
39–40	8641/8642	Tennessee Rex	1F:1M	Rexoid hair coat	*Unknown*
41	6623	Oriental	M	Lymphoma	*Unknown*
41		14 breeds	19F:18M:4U	~ 31 diseases and traits	

A complete description of diseases and traits for entire cohort. Candidate genes are potential genes that been identified with less evidence of a causal mutations.

*U* unknown sex, *F* female, *M* male.

^a^Mutations as tentative causal variants for diseases presented.

### Sequence coverage and specificity

**Figure 1:**
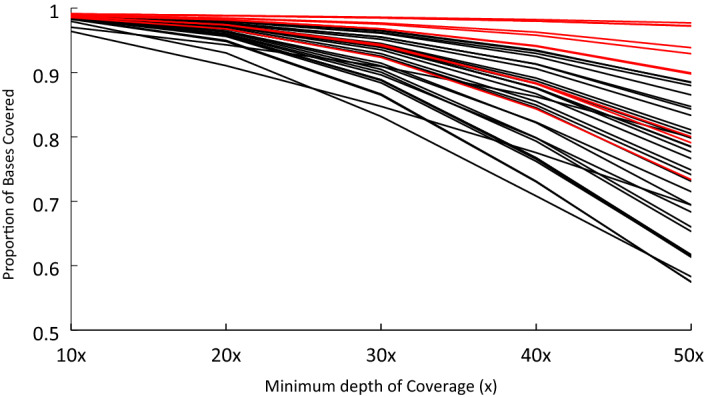
The proportion of bases covered with the exome capture probes. The initial 10 samples are colored in red, with the X axis showing the depth of coverage, which is how many times a nucleotide base is covered starting at a depth of  10x and increasing to 50x.

To assess the performance of this feline exome resource, deep coverage WES data was produced for ten cats with WGS data for comparison. After mapping to Felis_catus_9.0, base quality trimming, and PCR duplicate removal, the average percentage of reads uniquely mapped was 82% (Table 2). The average sequencing depth was 267 × with a range of 76 × to 458 × (Supplementary Table 2, Supplementary Data S1). Assessing the depth of coverage, of the 201,683 exonic targets, 98.1% aligned with coverage of > 20 ×. An average of 6.98% of the total reads aligned outside of the targeted regions of the genome. (Supplementary Table 2, Supplementary Data S1). For the uncharacterized 31 cat exomes, the sequencing depth was adjusted to typical human WES studies; for this group of cats, we estimated the average depth of coverage to be 80 ×. 96.41% of exonic targets aligned with a coverage of > 20 ×, ranging from 91 to 98%. An average of 10.41% of total reads aligned off-target is slightly higher when compared to the first 10 higher-coverage cats that can be attributed to lower sequencing depth in the larger cohort. As expected, overall there is a reduction in mapping at lower depth of coverage; for example, at 40 ×, 93.5% of targeted bases were covered (Fig. 1), conversely, 99% are covered at 2 ×.

**Table 2:** Summary of Metrics across both cohorts.

	Average-First 10	Range-First 10	Average-Cohort of 31	Range-Cohort of 31
Depth of coverage	267 ×	76–485 ×	80 ×	60–108 ×
% of bases covered	99.1%	92.3–100%	96.4%	91–98%
% reads aligned	99.9%	99.9–100%	82%	75–85%

### Platform-based variant discovery and associated biases

**Figure 2:**
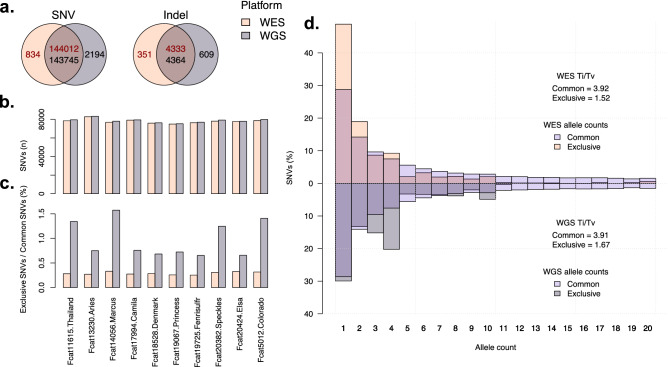
Variant calling statistics for 10 cats sequenced on both platforms. (**a**) Venn diagrams showing the number of exclusive and common variants per platform. Dark red text indicates the number of variants found in WES and black text indicates the number of variants found in WGS. The reason the number of common variants differ between platforms is because common variants were identified prior to filtering. (**b**) The number of SNPs found in each sample in both platforms. (**c**) The percentage of SNPs found as exclusive to each sample for each platform. The first, third, eighth, and tenth samples are males. All other samples are female. (**d**) Allele count distribution for common and exclusive SNPs in both platforms. WES SNPs are shown on top and WGS SNPs are shown upside down on the bottom. In addition, the Ti/Tv ratio for sets of SNPs is also shown.

Variants were divided into those found using both WES and WGS platforms and those exclusive to one platform. Both sets were then filtered for quality, variant type (SNV or indel), and biallelic status. For high impact variants causing a loss of function in the gene, WES and WGS identified 582 and 617 SNVs, respectively, with 97.8% of the WES SNVs also identified by WGS and 92.1% of the WGS SNVs also identified by WES (Table 3). The most common classes of variants identified exclusively by WGS were splice donor/acceptor sites and stop gains; however, the overall count of these variants was low, ranging from 3 to 19 total variants. Moderate (missense) and low (synonymous) impact variants had high concordance between the WES and WGS datasets, ranging from 94.7% for 3′ untranslated region SNVs in WGS to 100% for most SNVs identified by WES (Table 4). Altogether, only a small fraction of SNVs (WES = 834 and WGS = 2194) were exclusive to a particular platform (Fig. 2a). Considering indels identified by haplotype caller, the WES and WGS data had lower concordance than SNVs (Table 5). Although WES detected 1739 high impact indels and WGS detected 1931, the percentage of commonly identified and exclusive indels showed more variation between consequence categories than SNVs. For both SNVs and indels, those classified as high impact represented a disproportionate number of the platform exclusive variants. Across both platforms, each individual cat carried a total of approximately 80,000 SNVs within the exome target regions (Fig. 2b). As for platform exclusive SNVs, WGS SNV counts were elevated compared to WES SNV counts and also showed higher levels of variability between individuals (Fig. 2c).

**Table 3:** Indel consequence counts of WES versus WGS as determined by variant effect predictor.

Impact	Consequence	WES (%)			WGS (%)		
		Common	Exclusive	Total	Common	Exclusive	Total
High	Frameshift	1440 (93)	109 (7)	1549	1451 (84.8)	260 (15.2)	1711
High	Splice acceptor	69 (83.1)	14 (16.9)	83	71 (69.6)	31 (30.4)	102
High	Splice donor	107 (88.4)	14 (11.6)	121	107 (81.1)	25 (18.9)	132
High	Start lost	11 (100)	0 (0)	11	11 (84.6)	2 (15.4)	13
High	Stop gained	16 (76.2)	5 (23.8)	21	17 (56.7)	13 (43.3)	30
High	Stop lost	12 (92.3)	1 (7.7)	13	12 (85.7)	2 (14.3)	14
High	All	1602 (92.1)	137 (7.9)	1739	1615 (83.6)	316 (16.4)	1931
Moderate	Inframe deletion	709 (90.5)	74 (9.5)	783	710 (91.1)	69 (8.9)	779
Moderate	Inframe insertion	557 (92.4)	46 (7.6)	603	557 (90)	62 (10)	619
Moderate	Protein altering	13 (81.3)	3 (18.8)	16	13 (54.2)	11 (45.8)	24
Moderate	All	1267 (91.2)	122 (8.8)	1389	1268 (90.1)	139 (9.9)	1407
Low	3′ UTR	173 (91.5)	16 (8.5)	189	176 (81.5)	40 (18.5)	216
Low	5′ UTR	194 (96.5)	7 (3.5)	201	195 (91.5)	18 (8.5)	213
Low	Splice region	641 (94.8)	35 (5.2)	676	644 (92.9)	49 (7.1)	693
Low	Start retained	7 (100)	0 (0)	7	7 (100)	0 (0)	7
Low	Stop retained	10 (100)	0 (0)	10	10 (83.3)	2 (16.7)	12
Low	All	299 (94.3)	18 (5.7)	317	302 (92.9)	23 (7.1)	325
	All	4333 (92.5)	351 (7.5)	4684	4364 (87.8)	609 (12.2)	4973

**Table 4:** SNV consequence counts of WES versus WGS as determined by variant effect predictor.

Impact	Consequence	WES (%)			WGS (%)		
		Common	Exclusive	Total	Common	Exclusive	Total
High	Splice acceptor	97 (97)	3 (3)	100	98 (89.9)	11 (10.1)	109
High	Splice donor	137 (97.9)	3 (2.1)	140	139 (88)	19 (12)	158
High	Start lost	63 (96.9)	2 (3.1)	65	63 (100)	0 (0)	63
High	Stop gained	237 (97.9)	5 (2.1)	242	232 (92.8)	18 (7.2)	250
High	Stop lost	35 (100)	0 (0)	35	36 (97.3)	1 (2.7)	37
High	All	569 (97.8)	13 (2.2)	582	568 (92.1)	49 (7.9)	617
Moderate	missense	43,518 (99.3)	309 (0.7)	43,827	43,419 (98.1)	821 (1.9)	44,240
Moderate	All	43,516 (99.3)	309 (0.7)	43,825	43,417 (98.1)	821 (1.9)	44,238
Low	3′ UTR	2022 (97.9)	43 (2.1)	2065	2031 (94.7)	114 (5.3)	2145
Low	5′ UTR	2458 (99.5)	13 (0.5)	2471	2459 (98.6)	35 (1.4)	2494
Low	Splice region	3938 (99.5)	21 (0.5)	3959	3923 (98.7)	50 (1.3)	3973
Low	Stop retained	60 (100)	0 (0)	60	58 (96.7)	2 (3.3)	60
Low	Synonymous	87,341 (99.6)	321 (0.4)	87,662	87,182 (98.9)	956 (1.1)	88,138
Low	All	88,584 (99.6)	336 (0.4)	88,920	88,417 (98.9)	975 (1.1)	89,392
All	All	144,012 (99.4)	834 (0.6)	144,846	143,745 (98.5)	2194 (1.5)	145,939

**Table 5:** Mean SNVs per individual for ten WES and WGS cats.

Genes	Top 50 WGS outliers					
Platform	WGS			WES		
Sex	Male	Female	Difference (%)^a^	Male	Female	Difference (%)^a^
Autosome	1595.00	1445.67	149.33 (9.36)	946.25	872.83	73.42 (7.76)
X chromosome	1363.75	22.83	1340.92 (98.33)	829.75	23.00	806.75 (97.73)

Genes	All					
Platform	WGS			WES		
Sex	Male	Female	Difference (%)^a^	Male	Female	Difference (%)^a^
Autosome	53,724.75	57,605.50	3880.75 (7.22)	53,189.50	57,217.67	4028.17 (7.57)
X chromosome	1968.50	766.00	1202.50 (61.09)	1412.00	776.33	635.67 (45.02)

^a^Percentage differences in parentheses were calculated as a fraction of mean SNVs per male individual.

Another method for characterizing platform exclusive SNVs is to measure their allele count distributions. WES exclusive SNV allele counts were heavily skewed towards allele counts of one (Fig. 2d). Using SNVs found in both platforms as a standard for comparison, the WES exclusive allele count distribution is consistent with SNVs identified by random error, as most of these SNVs only appear once in the dataset. Moreover, this result is reflected by the Ti/Tv ratios of each dataset, the proportion of transitions to the number of transversions, which is used as a quality indicator for SNVs. WES SNVs found in both platforms have a Ti/Tv ratio of 3.92, indicating a low concentration of false-positive variant sites, while WES exclusive SNVs have a ratio of 1.52, indicating a high concentration of false-positive variant sites. Alternatively, allele counts for WGS exclusive SNVs have two peaks. The first is at an allele count of one, which is similar to WES exclusive SNVs, and the second is at an allele count of four, which is suggestive of more systematic error in variant detection. This second peak for WGS exclusive SNVs is likely consistent with the increased WGS exclusive variant detection observed in male cats and may be suggestive of issues stemming from the lack of a Y chromosome within the reference assembly that was used. For WGS SNVs, the Ti/Tv ratios for both exclusive and non-exclusive SNVs is similar to WES SNVs, where exclusive SNVs are enriched for false-positive variant sites.

**Figure 3:**
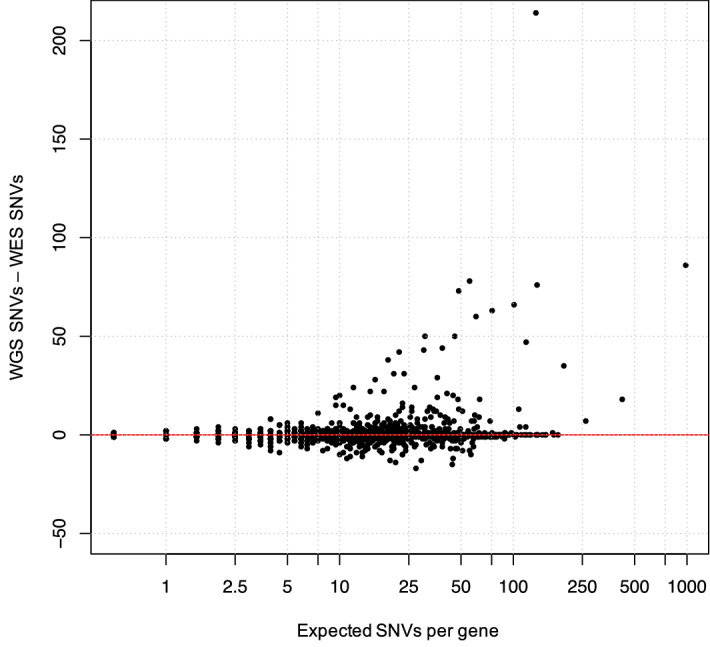
Gene-wise platform bias. Each individual point on the scatterplot is a gene with the y axis displaying differences in SNP counts per gene. Genes with more WGS SNPs than WES SNPs have positive values, where genes have negative values when there is more WES SNPs instead. Expected SNP number is calculated as the mean number of SNPs per gene across both platforms and is plotted on a log scale.

To detect bias toward specific genes using the WGS and WES platforms, the number of variants per gene was compared between WGS and WES results (Supplementary Data S2). When comparing variants discovered by WGS and WES, a large number of genes contained 20 or more variants discovered by WGS (Fig. 3). To investigate the cause for these outliers, the top 50 outlier genes were selected for further analysis (Supplementary Data S3). Of these, 14 genes were found on the X chromosome, suggesting differences in variant detection may correspond to the increased number of WGS exclusive SNVs in males observed in Fig. 2c (Supplementary Data S3). Apart from enrichment on chromosome X, another cluster of 13 genes with WGS-biased variant detection was located on chromosome D1. These genes were mostly olfactory receptors, which generally belong to large gene families with many paralogues and pseudogenes, likely leading to increased off-target effects. Another gene of note, LOC101099449, contained 713kbp of the target sequence. When analyzed more closely, LOC101099449’s target sequence overlapped an entire Immunoglobulin lambda locus, a region that is usually highly variable between individuals. All other genes with WGS-biased variant detection were distributed throughout the genome.

To further investigate increased WGS-biased variant detection on chromosome X, the mean number of variants per individual was compared between males and females (Table 5). Across autosomes and sequencing platforms, sex-based percentage differences were relatively low, ranging between 7 and 10%. Alternatively, across both gene groupings, the percentage difference between the sexes on the X chromosome were much higher. For the top 50 WGS outlier genes, both platforms showed an approximate 98% sex difference, whereas all genes showed a 61% sex difference for WGS and a 45% sex difference for WES. Since the percentage sex difference in outlier genes is similar across both platforms, results suggest that platform bias on chromosome X is more likely due to platform exclusive increased variant detection in these regions, rather than differential abilities of platforms to detect variants in either sex. Importantly, the actual number of chromosome X sex differences in both platforms is similar across gene groupings. In the top 50 WGS outliers, the difference between the chromosome X mean male and female SNV counts is 1340.92, while across all X chromosome genes this same difference is equal to 1202.5 (Table 5).

**Figure 4:**
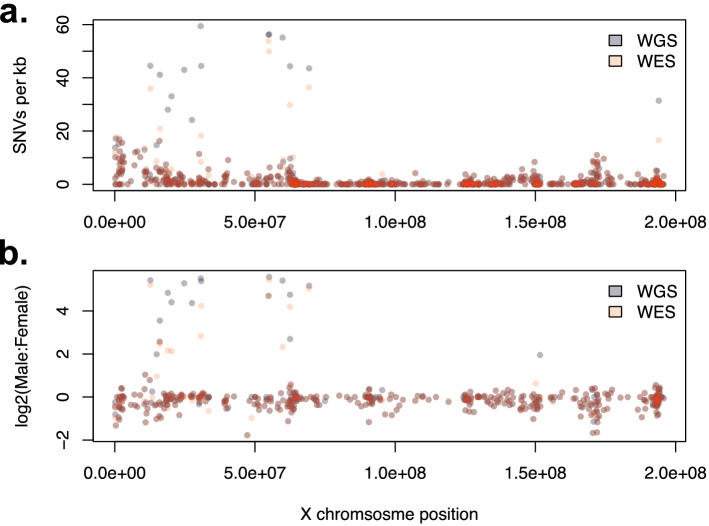
Distribution of SNPs per gene along chromosome X. (**a**) Total SNPs per kb of coding sequence per gene. (**b**) Sex biased variant detection along chromosome X. Bias is calculated as fold change ratio between the mean number of SNPs per individual per gene for males and females. Specifically, this was calculated for each gene as log2((mean male SNPs + 1)/(mean female SNPs + 1)). The ones were added to remove undefined results caused by dividing by the number 0.

To examine the potential overlap between platform and sex bias, the distribution of SNVs per gene along chromosome X were analyzed. Platform biased genes are clustered between positions 15 to 70 Mb (Fig. 4a). Across both platforms, these genes also have the highest SNV concentration, with > 20 SNVs per kb of coding sequence (Fig. 4a). Alternatively, the majority of genes outside this region have SNV concentrations of < 5 SNVs per kb of coding sequence. Regarding sex bias, while the overall percentage difference across platforms is similar (Table 5), individual genes show platform exclusive variability in effect size. For example, male biased variant detection on a per gene basis was observed more often for WGS (Fig. 4b). However, despite this variation across platforms, the genes with increased sex bias were the same genes with increased platform bias (Supplementary Data S4). Therefore, on chromosome X, platform biases and sex biases in SNV discovery appear confounded, as numerous factors within the same genes are relatively consistent across both platforms, both biases likely have a similar underlying root cause differently expressed in each platform.

A potential cause of sex bias in variant discovery is that the biased genes have degraded copies on the Y chromosome. For the ten known feline X chromosome genes with degraded Y copies^19^, the total number of SNVs per platform and the mean number of SNVs per individual were calculated. Of these ten genes, nine have more than 11 platform exclusive differences in SNV discovery and are therefore among the top 50 outlier genes for platform exclusive bias (Supplementary Table 4). Moreover, almost all SNVs found in these genes were found only in males, regardless of platform. For WGS there was an average total of 1169.25 SNVs found in males with only an average total of 7.83 found in females. For WES, the numbers were similar, with an average total of 774.5 SNVs found in males and an average total of 7.83 SNVs found in females (Supplementary Table 4). Together these results indicate a major portion of sex bias in variant discovery is due to the absence of a Y chromosome in the Felis_catus_9.0 assembly.

### Known variant validation

To further analyze the effectiveness of WES for variant detection, we examined each sample for the presence of known trait-causing variants. The Felis_catus_9.0 Ensembl release 99 gene annotation was used with a selection of exons with + /− 30 bp to match exome capture design and variants were browsed using the VarSeq software (GoldenHelix, Inc). The majority of the previously published 115 trait causing variants in the domestic cat that have been documented as causal for diseases and traits affect either the coding regions or a splice donor/acceptor site^16^. Of these known variants, 44 were identified in our WES cohort. All variants for coat colors and diseases expected to be present in the ten cats were identified, including the alleles in the loci for Agouti (*ASI*P—a^20^), Brown (*TYRP1*—b^21^), Color (*TYR*—cs^22^), Dense (*MLPH*—d^23^), Longhair (*FGF5*—I^24^), Lykoi (*HR*—hrTN, hrVA^25^), Bengal progressive retinal degeneration (*KIF3B*^26^) and Persian progressive retinal degeneration (*AIPL1*^17^), hydrocephalus (*GDF7*^27^), and others (Supplementary Data S5). The cats also had variants known to affect cat blood type as well^28,29^. In accordance with the limitations of our feline exome capture design, neither known structural nor intronic variants were detected. When analyzing discordant reads in a WGS dwarf sample, a deletion and rearrangement indicating a structural variant (SV) was visible in the *UDGH* gene^17^, but no read discordance was found in the WES analysis (Supplementary Fig. 1). In addition, the *KIT* intron one SV for White and Spotting were not identified^30^. Therefore, the WES approach will fail to identify many complex SVs, an important limitation to consider for future feline trait discovery efforts.

### Novel candidate variant discovery

Novel DNA variants were explored as putatively causal for diseases and traits in 33 cats. A novel frameshift mutation in polycystin 2 (*PKD2*^31^), a gene associated with polycystic kidney disease (PKD) was predicted to disrupt protein function in a Siberian cat shown by ultrasound to have PKD. This mutation, a single-base deletion, causes a truncated protein (p.Lys737Asnfs*2). This variant was heterozygous in the affected cat and unique to the exome data and was not identified in the 195-cat cohort of the 99 Lives variant dataset^32^. This variant was also identified in both grandparents on the dam’s side of the pedigree, although kidney ultrasound was not available. However, analysis of other Siberian cats with PKD diagnosed by ultrasound failed to identify the c.2211delG variant in *PKD2*, suggesting that this could be a private variant and that other disease-causing PKD variants are yet to be discovered in this breed.

A variant in the lysophosphatidic acid receptor 6 (*LPAR6*) gene associated with the autosomal recessive rexoid (Marsella wave) coat of the Cornish rex breed was detected in a Peterbald cat, which is a hairless breed^33^. However, the hairless trait is considered autosomal dominant by cat breeders. The annotation predicts a c.249delG causing a p.Phe84Leufs*10; therefore, this Peterbald cat likely is compound heterozygous for two mutations juxtaposed in LPAR6. This variant was heterozygous in the affected cat, unique to the exome data and not identified in the 99 Lives variant dataset.

A known feline disease variant was also re-identified (Supplementary Data S5)^32^. A solute carrier family 3-member 1 (*SLC3A1*) variant was homozygous in a Greek cat presenting with cystinuria. The c.1342C>T variant, causing a p.Arg448Trp at position A3:66539609 has been previously documented to be associated with this condition^34^. No other cat in the exome dataset had this variant. Many of the variants associated with cat blood group B and its extended haplotype were detected in 11 cats, suggesting five cats as type B, one was confirmed^28^. Variants were detected in *APOBEC3*, which is associated with feline immunodeficiency virus (FIV) infection in cats, and three cats had the allelic combination producing the IRAVP amino acid haplotype that is associated with FIV resistance^35^. Novel findings included two cats that were heterozygous for a porphyria variant in UROS (c.140C>T, c.331G>A)^36,37^, one cat which was homozygous for FXII deficiency variant (FXII_1631G>C)^36^, and had died as a kitten, and one cat which was heterozygous for a copper metabolism deficiency in *ATP7B*^38^. Additional variants for neuronal ceroid lipofuscinosis, pycnodysostosis, Ehlers-Danlos syndrome, hypothyroidism, and hypovitaminosis D, and several individual-specific variants for hypertrophic cardiomyopathy are under further investigation (Table 1).

## Discussion

In humans, WES has flourished over the past few years and is becoming more common in the practice of genomic medicine, especially newborn screening^39^. This is not currently the case for veterinary medicine due to several factors: a dog or cat owner’s unwillingness to incur the costs, lower accuracy of available genome references^40^, and the uncertainty of treatment options driven by sequence variant data. Well-annotated genomes and extensive resources, such as for human and mouse, have led to the development of various exome capture products ranging from those with a very limited focus, e.g., oncogene panels, to more extensive designs including 5′ and 3′ untranslated regions, predicted regulatory elements, and non-coding RNAs. For other mammals, exome capture designs have ranged from 44.6 Mb in pigs^41^ to 146.8 Mb in rats^42,43^, illustrating the variation in experimental objectives. In companion animals, only the domestic dog has exome capture probes available, which span 53 to 152 Mb with an overlap of 34.5 Mb between the capture designs^44,45^. In this study, a feline exome resource was developed by designing capture probes against the annotated Felis_catus_9.0 genome assembly, a highly contiguous assembly that enabled efficient probe design^40^. The targeted 35.7 Mb accounts for the exons and 30 bp of flanking sequences to minimize the loss of detectable splice donor and acceptor variants.

Success in disease variant identification in any species using WES is dependent on multiple factors, including mode of inheritance, sequencing depth, and efficient probe design that covers the regions of interest with high specificity, minimizing the number of off-target reads. Sequence coverage of ≥ 20 × is generally regarded as the standard to efficiently detect heterozygous variants^46^. At this threshold, an acceptable average target coverage of 96.4% was obtained in our study. In our first WES experiment of 10 cats, we achieve maximum exonic coverage of 99% with a mean depth of 267 × at aligned bases. However, we have found this high-depth approach is not necessary or cost-efficient for the discovery of feline associated disease variants. The first domestic dog exome design^44^, which covered 52.8 Mb distributed over 203,059 regions, had a range of 87–90% mapped reads at a 102 × mean sequencing depth. An updated canine design^44^ had 93.5% of the targeted bases (< 53 Mb) covered to at least 1X depth of coverage, while in our feline exome design, the on-target reads were nearly 100% at 10 × sequencing depth. Whilst absolute dog and cat exome comparisons are difficult due to the differences in annotation, genome assembly accuracy, and design techniques, both of these resources reveal acceptable performance.

The intended application of the cat WES was twofold: the identification of heritable, Mendelian diseases and traits, and somatic mutations in cancer. In this study, the focus was the former and included the assessment of the efficiency of the feline exome design for SNV discovery against ten matched WGS samples. The matched WGS and WES cats had an average of 30 × and 267 × depth of coverage, respectively, with the vast majority of SNVs and indels in overlapping regions being detected by both platforms. Altogether, these findings suggest the use of this feline exome probe set was extremely consistent with variant discovery from WGS, where 99.4% were uncovered in WGS while only 1.5% were absent from the WES cats. Consistent with large cohort human studies, indel discovery was less consistent (92.5% overlap) with 12.2% of WGS indels absent from WES data owing to the well-known short-read misalignment problem in regions with indels of varying size. Differences in the number of common variants between platforms is due to differential filtering, as common variants were identified prior to when filtering was performed. The percentage of exclusive variants per platform also varied according to variant impact, with high impact variants representing the largest percentage of exclusive variants for their impact class. Since high impact mutations are generally rare due to their impact on normal gene function, their enrichment within platform exclusive variant sets is expected. In the same manner, as low impact variants have no impact on gene function, they are less likely to be identified as platform exclusive within their variant class.

For a small number of genes, a larger number of SNVs was detected using WGS. These genes were mostly restricted to olfactory receptors on chromosome D1 and genes on the X chromosome that has degraded copies on the Y. The repetitive nature of olfactory receptors means they are likely to lead to a higher percentage of off-target reads, especially in pseudo-genes, and decrease mapping quality in legitimate targets. For X chromosome WGS biased genes, the increased variant discovery in males is likely a result of some genes residing in the degenerate X region of the Y chromosome. A collection of ten known X chromosome genes with diverging Y chromosome copies all showed high levels of sex and platform bias. These genes had more variants in males since the Y chromosome copies contained a large number of mismatches. Similarly, the divergence of the Y chromosome could affect the hybridization of Y sequences to X chromosome probes, leading to reduced detection of variants in WES cats. However, the number of variants in females for these genes was largely consistent across platforms, indicating that discrepancies are most likely due to the presence of the Y chromosome. The impact from degraded X genes on the Y chromosomes propagated throughout the analysis. WGS exclusive SNVs were more common in males and the allele count distribution contained a peak at an allele count of four. Even though male X chromosome carried more variants than female X chromosome across both platforms, the effect was especially observable in the WGS exclusive dataset and may have otherwise remained hidden without this comparison. Importantly, while the feline exome set contained probes for DDX3Y, USP9Y, UBE1Y, and KDM5D, which are all Y chromosome degraded X genes, these genes were not included in the reference genome used to align reads. Despite this absence of the partial Y assembly, many Y chromosomes degraded X genes do not have probes designed. Overall, both WGS and WES analysis of cat sex chromosomes will be improved by the assembly of a domestic cat Y chromosome.

Previously characterized and unknown germline or somatic variants of clinical significance, the former often not identifiable without the parents, were investigated to confirm if each were identical or unique to genes associated with each disease or phenotype in prior studies. Known variants were first confirmed to validate the accuracy of the cat exome design for the following aesthetic traits: Agouti, Brown, Dense, Gloves, Dilution, Extension, Long, Lykoi, and hairless coat types^16^. In addition, disease variants were found in genes earlier shown to be candidate alleles in hydrocephalus^47^, hypertrophic cardiomyopathy^48^, and progressive retinal atrophy^17^. These results importantly validate our design is capable of detecting variants with prior trait association. Nonetheless, a primary study objective was to find new potential causal variants in our small mixed disease and trait cohort of 31 domestic cats. This cohort was searched to find novel candidate variants for three diseases and traits; feline autosomal dominant polycystic kidney disease (ADPKD), atrichia, hypotricha. ADPKD is a common inherited autosomal dominant disease affecting about 6% of the world’s cats^49^ and is characterized by fluid-filled cysts that form in the bilateral kidneys that often leads to renal failure^50^. Many of the features of feline ADPKD are similar to human ADPKD and recent studies demonstrated the utility of the cat model^14,51^. The c. 10063C>A mutation in exon 29 of *PKD1* was the only known causative allele for feline ADPKD^49^, however, for human ADPKD, variants are found throughout PKD1. A variant in polycystin 2 (*PKD2*), c.2211delG at position B1:134992553, causes a p.Lys737Asnfs*2 and was identified in a Siberian cat from Europe, indicating additional alleles may be segregating for ADPKD in cats.

Domestic cats have various forms of atrichia and hypotricha, which even though each is characterized by baldness or loss of hair coat, are not considered diseased cats since breeders have selected upon these observed traits to develop new breeds. Only two breeds are recognized as completely hairless, the Sphynx and Donskoy. Donskoy cats are a breed of Russian cats in which loss of hair is determined by a semi-dominant allele^52^. Peterbald cats were bred in Russia in 1994 as a product of a Donskoy and an Oriental Shorthair cross, and are often born with no hair, or lose their hair over time^53^. Cornish Rex, a hypotrichia breed, that is characterized by a curly coat, is caused by a homozygous deletion mutation in *LPAR6*^54^. The Peterbald cat had an *LPAR6* 4 base pair deletion that is in juxtaposition to a compound heterozygote for the Cornish rex deletion variant. Both variants result in premature stop codons a few amino acids downstream of the variant site. Other disease-associated variants were re-identified, such as cystinuria variants, in which the cat was homozygous and affected. Determination of allele frequencies through the 99 lives project^40^ improved the identification of cats that were heterozygous for variants associated with recessive diseases, such as, porphyria^36^, Factor XII deficiency^55^, and copper metabolism^38^. The inclusion of 99 Lives WGS data was central to establishing the likelihood of variants being causal for diseases and further cross-species explorations of variant frequencies promises to better define variants of uncertain significance^56^.

Clinical use of sequence variant information in companion animals is in the very early stages, which hampers the ability of veterinarians to rapidly diagnose some diseases without standard or unclear phenotypic determinants. In the future, it could be used to adapt treatments to the specific animal and disease type^57^. Many diagnosed rare diseases have a poor prognosis, with some less than 90 days; thus, cost-effective sequencing approaches may help discover alternate and more effective treatments. The Undiagnosed Diseases Program of the National Institutes of Health routinely uses WES for this purpose of finding treatments where none exist, suggesting veterinary medicine could benefit in the same manner^58^. We confirm here, as other studies have shown, that WES is cost-effective, data process-efficient (by requiring less computing time), and easier to use than WGS for inferring a variant’s biological relevance^59^. As in the dog, a first step is offered toward the use of feline WES for robust disease variant detection, including the validation of previously identified causal alleles and the discovery of novel candidate variants that we suggest are of interest for further experimental scrutiny^60^. We have developed domestic cat-specific WES, and importantly, based on our findings, validated its use for the evaluation of potential disease variants for the future practice of feline genomic medicine.

## Methods

### Exome design

The annotated exons from the Felis_catus_9.0 reference genome assembly were used as the basis to design the exome capture probes, incorporating the NCBI RefSeq release 92 annotation, containing 19,590 refGene names. The coding sequences (CDS) for the primary chromosomes were extracted and consolidated into a non-overlapping set of features, and repetitive probes were removed totaling 35,724,716 bases divided over 201,683 regions. Of those bases, only 395,115 bp are not covered directly or indirectly. GO functions for removed genes were olfactory genes or unidentifiable. Since Y chromosome genes are not represented in the Felis_catus_9.0 reference, a set of coding sequence features from the *Felis catus* Y chromosome genomic sequence (NCBI accession KP081775) was used^61^. The cat exome panel was designed by Roche Sequencing Solutions (Madison, USA)^62^. A capture probe dataset was constructed for the full cat genome by tiling variable-length probes, ranging from 50 to100 bases in length, at a five-base step across all sequences. Each capture probe was evaluated for repetitiveness by constructing a 15-mer histogram from the full genome sequence and then calculating the average 15-mer count across each probe, a sliding window size of 15 bases across the length of each probe. Any probe with an average 15-mer count greater than 100 was considered to be repetitive and excluded from further characterization. Non-repetitive probes were then scored for uniqueness by aligning each capture probe to the full cat genome using SSAHA v3^63^. A close match to the genome was defined as a match length of 30 bases, allowing up to five insertions/deletions/substitutions. Capture probes were selected for each coding sequence feature by scoring one to four probes in a 20-base window, based on repetitiveness, uniqueness, melting temperature, and sequence composition, and then choosing the best capture probe in that window. The start of the 20 base windows was then moved 40 bases downstream and the process repeated. Selected probes were allowed to start up to 30 bases before the 5′ start of each feature and overhang the 3′ end by 30 bp. A maximum of five close matches in the genome was allowed when selecting the capture probes.

### Samples and DNA isolation

Cat DNA samples for WES were donated by owners and archived in accordance with the University of Missouri Institutional Animal Care and Use Committee protocol study protocols 9056, 9178, and 9642. DNA was isolated from 41 whole blood or tissue cat samples using standard organic methods^64^ and verified for quantity and quality by DNA fluorescence assay (Qubit, Thermo Fisher) and ethidium bromide staining after 0.7% agarose gel electrophoresis. Ten cats with existing whole genome sequence (WGS) data were initially tested, followed by 31 novel cats.

### Sequencing

All WGS cat data used in this study was obtained from Beuckley et al.^32^ Genomic DNA (250 ng) was fragmented on the Covaris LE220 instrument targeting 250 bp inserts. Automated dual indexed libraries were constructed with the KAPA HTP library prep kit (Roche) on the NGS platform (Perkin Elmer). The libraries were PCR-amplified with KAPA HiFi for 8 cycles. The final libraries were purified with a 1.0 × AMPureXP bead cleanup and quantitated on the Caliper GX instrument (Perkin Elmer) and were pooled pre-capture generating a total 5 µg library pool. Each library pool was hybridized with a custom NimbleGen probe set (Roche), targeting 35.7 Mb. The libraries were hybridized for 16–18 h at 65 °C followed by washing to remove non-specific hybridized library fragments. Enriched library fragments were eluted following isolation with streptavidin-coated magnetic beads and amplified with KAPA HiFi Polymerase prior to sequencing. PCR cycle optimization was performed to prevent over-amplification of the libraries. The concentration of each captured library pool was determined via qPCR utilizing the KAPA library Quantification Kit (Roche) to produce appropriate cluster counts prior to sequencing. The Illumina NovaSeq6000 instrument was used to generate paired-end 2 × 150 bp length sequences to yield an average of 14 Gb of data per 35.7 Mb target exome, producing ~ 80 × exome sequencing depth of coverage. Exome sequencing data are available at the Sequence Read Archive under accession number PRJNA627536.

### Variant discovery

The following tools/packages were applied to WGS and WES samples in accordance with variant processing as previously described^32^ 71: BWA-MEM version 0.7.17^65^, Picard tools version 2.1.1 (http://broadinstitute.github.io/picard/), Samtools version 1.9^66^, and Genome Analysis toolkit version 3.8^67–69^. Code used for the variant calling workflow can be found at https://github.com/mu-feline-genome/batch_GATK_workflow. For WES processing, GATK tools were restricted to exons annotated in Ensembl release 99 with an additional 100 bp of flanking sequence^70^. Following processing, samples were genotyped in three separate cohorts. The first cohort consisted of all 41 WES samples. The second and third cohorts were ten matched WES and WGS samples. Variants in all three cohorts were tagged using the same variant filtering criteria. For SNVs, the filtering criteria were QD < 2.0, FS > 60.0, SOR > 3.0, ReadPosRankSum < − 8.0, MQ < 40.0, and MQRankSum < − 12.5. For indels, the filtering criteria were QD < 2.0, FS > 200.0, SOR > 10.0, and ReadPosRankSum < − 20.0. Although five Y chromosome genes were included in the exome probe set, these genes had not been added to the aligning reference. For WGS/WES comparison, matched WES/WGS samples were annotated using variant effect predictor release 97(VEP)^71^. Variants from both cohorts were independently tagged as to whether they were biallelic, SNVs, or passed filtering criteria. Before analysis, variants flanking the exome primary target regions + /− 2 bp were removed (Supplementary Data S1). Variant processing and comparisons were performed in the R statistical environment using the vcfR package version 1.8.0^72^. Common variants between both platforms were determined as those at the same position with the same reference and alternate alleles. Exclusive variants were determined as those where the position and/or the alleles were specific to a particular platform.

### Disease and trait variant detection

Variants for all 41 cats were evaluated using VarSeq software (GoldenHelix, Inc.). SNVs were annotated as having high, moderate, or low impacts on gene function. High impact variations were those that were a protein-truncating variant caused by stop gain or loss and splice-site acceptor or donor mutations^73^. Moderate impacts include missense mutations or in-frame insertions, and lastly, low impact variants are characterized by synonymous base changes, splice region variants, or intron variants. Known variants for diseases and traits were evaluated in each cat.

### Polycystic kidney disease

A pointed cat of the Siberian breed (a.k.a. Neva Masquerade, a pointed Siberian) was diagnosed with polycystic kidney disease based on signs of renal disease (polydipsia, polyuria) and ultrasonography (Table 1, cat 37). DNA was submitted using buccal swabs and a whole blood sample to two different commercial testing laboratories in which both confirmed the absence of the currently known autosomal dominant polycystic kidney disease in polycystin-1 (PKD1)^49,74^. The dam and a sibling were also reported as having PKD by ultrasonography but were not available for genetic analyses.

### Cystinuria

A 3-month-old European shorthair kitten from the isle of Korfu, Greece, was presented to the AniCura Small Animal Hospital, Bielefeld, FRG, for heavy straining during urination, and the owner reported the kitten would fall over from time to time (Table 1, cat 17). The kitten had been pretreated with two injections of cephalexine and dexamethasone for suspected cystitis, however, difficulty in urination worsened. Upon hospital admission, the kitten was in good general condition. Abdominal palpation revealed an enlarged urinary bladder. Abdominal X-ray showed over 30 radiolucent urinary stones up to a diameter of half of the width of the last rib. Urinary bladder stones and some urethral stones were removed via cystolithotomy and retrograde flushing of the urethra. Urinary stones were submitted for infraspectroscopic stone analysis. Stone analysis revealed pure cystine stones and a diagnosis of cystinuria was made. Urinary stones reoccurred at 6 months of age, but the kitten was otherwise healthy.

### Informed consent

Cat DNA samples for WES were donated by owners. The study protocol was approved by the University of Missouri Institutional Animal Care and Use Committee protocol study protocols 9056, 9178, and 9642. All experiments were performed in accordance with relevant guidelines and regulations. Informed consent was also obtained from owners for involvement for animals in our study.

## Supplementary Information

Supplementary Information 1.

Supplementary Information 2.

Supplementary Information 3.

Supplementary Information 4.

Supplementary Information 5.

Supplementary Information 6.

## Data Availability

The code for probe design is not available. Roche Sequencing solutions has HyperExplore panels available with the KAPA Target Enrichment platform for probe design.
