# Supplementary material for: A domestic cat whole exome sequencing resource for trait discovery
Source: Sci Rep. 2021 Mar 30;11:7159. doi: 10.1038/s41598-021-86200-7 (PMC8009874; doi:10.1038/s41598-021-86200-7)
Supplement: Supplementary file 1 — Supplementary Information 1. [file 41598_2021_86200_MOESM1_ESM.docx]

**A domestic cat whole exome sequencing resource for trait discovery**

Alana R. Rodney^1,*^, Reuben M. Buckley^2,*^, Robert S. Fulton^3^, Catrina Fronick^3^, Todd Richmond^4^, Christopher R. Helps^5^, Peter Pantke^6^, Dianne J. Trent^7^, Karen M. Vernau^8^, John S. Munday^9^, Andrew C. Lewin^10^, Rondo Middleton^11^, Leslie A. Lyons^2^, Wesley C. Warren^1,†^.

^1^Department of Animal Sciences, College of Agriculture, Department of Surgery, School of Medicine, Institute for Data Science and Informatics, University of Missouri, Columbia, Missouri, 65211, USA

^2^Department of Veterinary Medicine & Surgery, College of Veterinary Medicine, University of Missouri, Columbia, Missouri, 65211, USA

^3^McDonnell Genome Institute, Washington University, School of Medicine, St Louis, Missouri, 63108, USA

^4^Roche Sequencing Solutions, Pleasanton, CA, 94588, USA

^5^Langford Vets, University of Bristol, Langford, Bristol, BS40 5DU UK

^6^AniCura Bielefeld GmbH, Tierärztliche Klinik für Kleintiere, Bielefeld, 33719, Germany

^7^Department of Biomedical and Diagnostic Sciences, College of Veterinary Medicine, University of Tennessee, Knoxville, Tennessee, 37996, US

^8^School of Veterinary Medicine, University of California Davis, Davis, California, 95616, USA

^9^School of Veterinary Science, Massey University, Palmerston North, New Zealand

^10^Department of Veterinary Clinical Sciences, Louisiana State University, Baton Rouge, Louisiana, 70803, USA

^11^Nestlé Purina Research, Saint Louis, Missouri 63164, USA

*Authors provided equal contribution

^†^Corresponding author: warrenwc@missouri.edu

Running title: **Feline exome sequencing resources**

Keywords: Animal models, feline, exome, *Felis catus*, WES, Precision Medicine

**Supplementary Files**

Supplementary Information: Supplementary Tables and Figures.

Supplementary Data S1: Exome primary targets

Supplementary Data S2: Platform bias of genes indicated as the difference in the total number of variants in each platform. Genes are sorted by WGS – WES variants, largest to smallest.

Supplementary Data S3: The top 50 genes from Supplementary Data S2 sorted by position.

Supplementary Data S4: Sex bias of all X chromosome genes. Columns represent the mean number of SNVs per individual for a particular platform and sex. For example, “WGS.m” is the mean number of WGS variants per male individual.

Supplementary Data S5: Confirmed known variants

**Supplementary Table 1. Summary of metrics across all cats.**

| Cat | **TOTAL READS** | **Unique Reads** | **% Unique Reads** | **Mapped Unique Reads** | **% Unique Reads Mapped** | **MEAN COVERAGE** | **MEDIAN COVERAGE** |
| --- | --- | --- | --- | --- | --- | --- | --- |
| 1 | 66980388 | 53730990 | 80.22% | 53700410 | 99.94% | 87 | 79 |
| 2 | 55650338 | 45654534 | 82.04% | 45633063 | 99.95% | 75 | 69 |
| 3 | 145242280 | 118605692 | 81.66% | 118567772 | 99.97% | 199 | 181 |
| 4 | 83764798 | 68089930 | 81.29% | 68065004 | 99.96% | 112 | 103 |
| 5 | 164681954 | 134926578 | 81.93% | 134884666 | 99.97% | 23 | 200 |
| 6 | 64431286 | 52495816 | 81.48% | 52473634 | 99.96% | 82 | 75 |
| 7 | 100048280 | 81109770 | 81.07% | 81072154 | 99.95% | 133 | 120 |
| 8 | 98788892 | 78294144 | 79.25% | 78264012 | 99.96% | 133 | 122 |
| 9 | 150859656 | 120941838 | 80.17% | 120901295 | 99.97% | 203 | 185 |
| 10 | 82184788 | 67558538 | 82.20% | 67528042 | 99.95% | 115 | 105 |
| **Mean** | 101263266 | 82140783 | 81.13% | 82109005.2 | 99.96% | 137.5 | 123.9 |
| 11 | 65352134 | 52724620 | 80.68% | 52631834 | 99.82% | 83.807697 | 76 |
| 12 | 61091136 | 49625330 | 81.23% | 49566582 | 99.88% | 77.745496 | 69 |
| 13 | 46864618 | 39837652 | 85.01% | 39789028 | 99.88% | 63.66044 | 57 |
| 14 | 57105380 | 45115204 | 79.00% | 45076149 | 99.91% | 62.204826 | 57 |
| 15 | 73192396 | 59937688 | 81.89% | 59848302 | 99.85% | 94.199251 | 85 |
| 16 | 78645830 | 63544212 | 80.80% | 63467660 | 99.88% | 105.730205 | 95 |
| 17 | 49270872 | 41010968 | 83.24% | 40948904 | 99.85% | 63.033586 | 57 |
| 18 | 51213246 | 42192148 | 82.39% | 42135615 | 99.87% | 66.988664 | 61 |
| 19 | 62943656 | 49742358 | 79.03% | 49665648 | 99.85% | 80.385844 | 73 |
| 20 | 49270872 | 41010968 | 83.24% | 40948904 | 99.85% | 63.033586 | 57 |
| 21 | 66562284 | 55076186 | 82.74% | 54994060 | 99.85% | 90.477224 | 82 |
| 22 | 65035570 | 54927188 | 84.46% | 54853614 | 99.87% | 87.959322 | 80 |
| 23 | 72513806 | 56798782 | 78.33% | 56735443 | 99.89% | 90.677266 | 83 |
| 24 | 57911238 | 48615406 | 83.95% | 48535472 | 99.84% | 78.836957 | 71 |
| 25 | 82008038 | 64579700 | 78.75% | 64483066 | 99.85% | 108.154842 | 97 |
| 26 | 92835910 | 72587612 | 78.19% | 72499058 | 99.88% | 103.520067 | 94 |
| 27 | 59689994 | 45040110 | 75.46% | 44947330 | 99.79% | 73.46272 | 65 |
| 28 | 57055464 | 47567866 | 83.37% | 47493680 | 99.84% | 75.851487 | 69 |
| 29 | 63399300 | 52257320 | 82.43% | 52181036 | 99.85% | 85.08411 | 77 |
| 30 | 72860408 | 60362784 | 82.85% | 60276637 | 99.86% | 97.22921 | 88 |
| 31 | 77857484 | 59696054 | 76.67% | 59618838 | 99.87% | 92.62662 | 84 |
| 32 | 53514212 | 44731352 | 83.59% | 44666286 | 99.85% | 75.052705 | 68 |
| 33 | 46245536 | 38645300 | 83.57% | 38588802 | 99.85% | 60.018781 | 54 |
| 34 | 66297558 | 55692274 | 84.00% | 55613569 | 99.86% | 88.7018 | 80 |
| 35 | 63195078 | 52759900 | 83.49% | 52685856 | 99.86% | 83.577563 | 76 |
| 36 | 61610752 | 49451286 | 80.26% | 49384858 | 99.87% | 78.57423 | 72 |
| 37 | 46067970 | 37352402 | 81.08% | 37288379 | 99.83% | 60.106841 | 54 |
| 38 | 61427656 | 47373818 | 77.12% | 47300394 | 99.85% | 77.19617 | 70 |
| 39 | 54045256 | 44167266 | 81.72% | 44066794 | 99.77% | 70.125402 | 64 |
| 40 | 49086732 | 40778946 | 83.08% | 40690449 | 99.78% | 66.461466 | 60 |
| 41 | 53287642 | 41298322 | 77.50% | 41236732 | 99.85% | 65.941935 | 57 |
| **Mean** | 79.6 | 58524991.31 | 81.23% | 58462334.2 | 99.88% | 89.76015 | 72 |

**Supplementary Table 2. Summary of base pairs covered across all cats**

| **Cat** | **Median Coverage** | **% target bp covered >10x** | **% target bp covered >20x** | **% target bp covered >30x** |
| --- | --- | --- | --- | --- |
| 1 | 79 | 99 | 100 | 103 |
| 2 | 69 | 99 | 99 | 98 |
| 3 | 181 | 99 | 98 | 97 |
| 4 | 103 | 99 | 99 | 99 |
| 5 | 200 | 99 | 100 | 101 |
| 6 | 75 | 99 | 97 | 94 |
| 7 | 120 | 100 | 101 | 104 |
| 8 | 122 | 99 | 97 | 92 |
| 9 | 185 | 99 | 99 | 100 |
| 10 | 105 | 100 | 101 | 105 |
| **Mean** | 123.9 | 99.2 | 99.1 | 99.3 |
| 31 | 76 | 98 | 95 | 86 |
| 26 | 69 | 98 | 95 | 87 |
| 41 | 57 | 98 | 93 | 83 |
| 34 | 57 | 98 | 96 | 88 |
| 35 | 85 | 98 | 96 | 89 |
| 20 | 95 | 98 | 96 | 89 |
| 36 | 57 | 98 | 96 | 89 |
| 30 | 61 | 98 | 96 | 90 |
| 12 | 73 | 98 | 96 | 90 |
| 29 | 57 | 98 | 96 | 91 |
| 11 | 82 | 99 | 96 | 90 |
| 25 | 80 | 98 | 97 | 92 |
| 40 | 83 | 99 | 96 | 91 |
| 37 | 71 | 98 | 97 | 93 |
| 32 | 97 | 99 | 97 | 93 |
| 21 | 94 | 96 | 91 | 85 |
| 13 | 65 | 99 | 97 | 94 |
| 16 | 69 | 99 | 97 | 94 |
| 28 | 77 | 99 | 97 | 94 |
| 39 | 88 | 99 | 97 | 94 |
| 24 | 84 | 99 | 97 | 94 |
| 15 | 68 | 99 | 97 | 94 |
| 27 | 54 | 99 | 97 | 94 |
| 14 | 80 | 99 | 98 | 95 |
| 33 | 76 | 99 | 98 | 96 |
| 17 | 72 | 99 | 98 | 95 |
| 18 | 54 | 97 | 94 | 91 |
| 38 | 70 | 99 | 98 | 96 |
| 23 | 64 | 99 | 98 | 97 |
| 19 | 60 | 99 | 98 | 96 |
| 22 | 57 | 99 | 98 | 96 |
| **Mean** | **72.00** | **98.45** | **96.41** | **91.84** |

**Supplementary Table 3: Platform and sex bias for X chromosome genes with degraded copies on the Y chromosome**

|  | **NCBI** | **Total SNVs** | | **WGS -** | **Mean WGS SNVs** | | **Mean WES SNVs** | |
| --- | --- | --- | --- | --- | --- | --- | --- | --- |
| **Gene pairs*** | **Gene** | **WGS** | **WES** | **WES** | **Male** | **Female** | **Male** | **Female** |
| UTX/UTY | *KDM6A* | 242 | 28 | 214 | 196.5 | 0.33 | 19.25 | 0.33 |
| EIF2S3X/EIF2S3Y | *EIF2S3* | 85 | 12 | 73 | 63.75 | 0 | 8.75 | 0 |
| ZFX/ZFY | *ZFX* | 107 | 44 | 63 | 98 | 0 | 42.75 | 0 |
| UBE1X/UBE1Y | *UBA1* | 142 | 95 | 47 | 96.5 | 0.5 | 64.5 | 0.5 |
| CXorf15/CYorf15 | *TXLNG* | 52 | 9 | 43 | 38.75 | 0.33 | 6.75 | 0.33 |
| JARID1C/JARID1D | *KDM5C* | 213 | 178 | 35 | 171 | 0 | 155 | 0 |
| EIF1AX/EIF1AY | *EIF1AX* | 19 | 0 | 19 | 16.75 | 0 | 0 | 0 |
| USP9X/USP9Y | *USP9X* | 433 | 415 | 18 | 360.75 | 6.5 | 362.75 | 6.5 |
| DDX3X/DDX3Y | *DDX3X* | 114 | 101 | 13 | 102.75 | 0.17 | 93.75 | 0.17 |
| AMELX/AMELY | *AMELX* | 26 | 21 | 5 | 24.5 | 0 | 21 | 0 |
| **Total** |  | **1433** | **903** | **530** | **1169.25** | **7.83** | **774.5** | **7.83** |

*As defined by Wilkerson et al., 2008.

**Supplementary Table 4: Platform and sex bias for degraded X genes on the Y chromosome**

|  | **NCBI** | **Total SNVs** | | **WGS -** | **Mean WGS SNVs** | | **Mean WES SNVs** | |
| --- | --- | --- | --- | --- | --- | --- | --- | --- |
| **Gene pairs*** | **Gene** | **WGS** | **WES** | **WES^1^** | **Male** | **Female** | **Male** | **Female** |
| UTX/UTY | *KDM6A* | 242 | 28 | 214 | 196.5 | 0.33 | 19.25 | 0.33 |
| EIF2S3X/EIF2S3Y | *EIF2S3* | 85 | 12 | 73 | 63.75 | 0 | 8.75 | 0 |
| ZFX/ZFY | *ZFX* | 107 | 44 | 63 | 98 | 0 | 42.75 | 0 |
| UBE1X/UBE1Y | *UBA1* | 142 | 95 | 47 | 96.5 | 0.5 | 64.5 | 0.5 |
| CXorf15/CYorf15 | *TXLNG* | 52 | 9 | 43 | 38.75 | 0.33 | 6.75 | 0.33 |
| JARID1C/JARID1D | *KDM5C* | 213 | 178 | 35 | 171 | 0 | 155 | 0 |
| EIF1AX/EIF1AY | *EIF1AX* | 19 | 0 | 19 | 16.75 | 0 | 0 | 0 |
| USP9X/USP9Y | *USP9X* | 433 | 415 | 18 | 360.75 | 6.5 | 362.75 | 6.5 |
| DDX3X/DDX3Y | *DDX3X* | 114 | 101 | 13 | 102.75 | 0.17 | 93.75 | 0.17 |
| AMELX/AMELY | *AMELX* | 26 | 21 | 5 | 24.5 | 0 | 21 | 0 |

*As defined by Wilkerson et al., 2008.

**Supplementary Figure 1.**


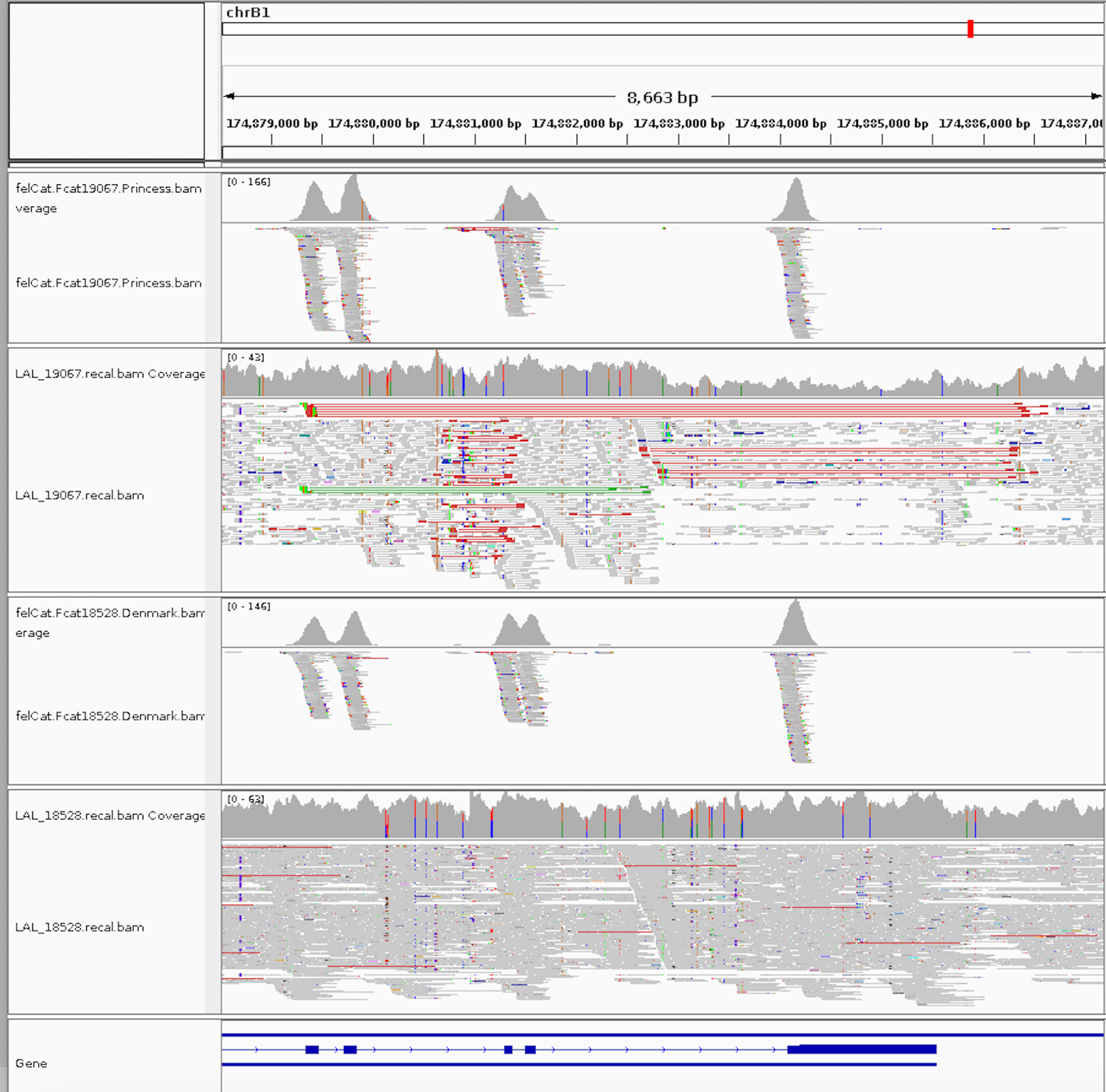
**Structural variants not covered by WES.** A visual showing reads that overlap the dwarfism structural variant in the dwarfism cat sample. The first row is the WES reads showing no evidence of a structural variant in the UDGH gene. Row 2 is the WGS reads showing a deletion and rearrangement (green and red). Thus, showing that WES does not adequately cover structural variants**.**
